# Supplementary material for: Cloud BioLinux: pre-configured and on-demand bioinformatics computing for the genomics community
Source: BMC Bioinformatics. 2012 Mar 19;13:42. doi: 10.1186/1471-2105-13-42 (PMC3372431; doi:10.1186/1471-2105-13-42)
Supplement: Additional file 1 — Supplementary 1 Cloud BioLinux software documentation in the form of a mini, self-contained website. Users need to download and uncompress the .zip file, and open through a web browser the "index.html" file available on the main directory. (ZIP 1823 kb). [file 1471-2105-13-42-S1.ZIP › Cloud-BioLinux-Package-Documentation/docs/stars.html]

Bio-Linux Software Documentation Pages

Back to search form

## stars

|  |  |
| --- | --- |
| Name | stars |
| Description | **stars** is an alternative interface to staden for studying polymorphisms in short sequences with batch processing, manual editing, trace viewing and data management.  Stars was initially designed for Multi Locus Sequence Typing of bacteria. |
| Homepage | http://sourceforge.net/projects/stars/ |
| Remote Documentation |  |
